# Identification of molecular correlations of RBM8A with autophagy in Alzheimer's disease

**DOI:** 10.18632/aging.102571

**Published:** 2019-12-09

**Authors:** Donghua Zou, Rongjie Li, Xiaohua Huang, Guoying Chen, Ying Liu, Youshi Meng, Yimei Wang, Yuan Wu, Yingwei Mao

**Affiliations:** 1 Department of Neurology, The Fifth Affiliated Hospital of Guangxi Medical University, Nanning, Guangxi 530022, China; 2 Department of Neurology, Affiliated Hospital of Youjiang Medical University for Nationalities, Baise 533000, China; 3 Department of Neurology, The First Affiliated Hospital of Guangxi Medical University, Nanning, Guangxi 530021, China; 4 Department of Biology, Pennsylvania State University, University Park, PA 16802, USA

**Keywords:** Alzheimer's disease, RBM8A, exon junction complex, neurodevelopment

## Abstract

Our previous studies revealed RBM8A may play a role in various progressive neurological diseases. The present study aimed to explore the role of RBM8A in Alzheimer's disease (AD). RBM8A is significantly down-regulated in AD. Interestingly, 9186 differentially expressed genes are overlapped from comparisons of AD versus control and RBM8A-low versus RBM8A-high. Weight gene correlation analysis was performed and 9 functional modules were identified. Modules positively correlated with AD and RBM8A-low are significantly involved in the RAP1 signaling pathway, PI3K−AKT signaling pathway, hematopoietic cell lineage, autophagy and APELIN signaling pathway. Fifteen genes (RBM8A, RHBDF2, TNFRSF10B, ACP1, ANKRD39, CA10, CAMK4, CBLN4, LOC284214, NOVA1, PAK1, PPEF1, RGS4, TCEB1 and TMEM118) are identified as hub genes, and the hub gene-based LASSO model can accurately predict the occurrence of AD (AUC = 0.948). Moreover, the RBM8A-module-pathway network was constructed, and low expression of RBM8A down-regulates multiple module genes, including FIP200, Beclin 1, NRBF2, VPS15 and ATG12, which composes key complexes of autophagy. Thus, our study supports that low expression of RBM8A correlates with the decrease of the components of key complexes in autophagy, which could potentially contribute to pathophysiological changes of AD.

## INTRODUCTION

More than 20 million people in the world suffer from a progressive neurodegenerative disorder, Alzheimer's disease (AD) [1, 2], which is the leading cause of dementia. The symptoms of the disease begin with mild memory difficulties and develop towards cognitive impairment, personality change and language impairment as the condition deteriorates [3]. So far, no effective treatment can cure AD. In addition, the long-term care of AD patients puts heavy economic burden to families and society. Epidemiological studies suggest that age, family history and the genetics are the three risk factors for AD [4–6]. Among them, age is the most important factor, most AD patients are over 65 years old [7, 8]. Moreover, cardiovascular disease, education, social and cognitive engagement and traumatic brain injury are also factors affecting the incidence of AD [9]. It has been found that genetic mutations of presenilin 1 (PSEN1), presenilin 2 (PSEN2), the epsilon 4 allele of the apolipoprotein E (APOE) and amyloid precursor protein (APP) on chromosomes 1, 14, 19 and 21 cause AD [10, 11].

In addition, several RNA-binding proteins (RNPs) have been identified to be strongly linked with neurodegenerative diseases, such as amyotrophic lateral sclerosis (ALS) [12, 13]. It is worth noting that other RNPs, including the exon junction complex (EJC), also have been found to play a prominent role in neurodevelopment [14]. EJC consists of four core proteins (EIF4A3, MAGOH, RBM8A and MLN51) [15, 16], of which RNA-binding motif protein 8A (RBM8A) is an RNP that is involved in nonsense mediated RNA decay (NMD) and RNA splicing [17, 18]. Studies have shown that high expression of RBM8A leads to increased anxiety-like behavior, abnormal social interaction and reduced immobile time [19]. Previously, we found that RBM8A regulates many genes related to neurodegenerative and neuropsychiatric disorders [20], but the role RBM8A in the progress of AD remains unclear.

To further explore the potential mechanism of R8M8A in AD, we conducted a comprehensive modular exploration. We found that the low expression of RBM8A may lead to the decrease of the components of key complexes (FIP200, Beclin 1, NRBF2, VPS15 and ATG12) in autophagy-pathway, which are associated with autophagy disorder and may contribute to the risk of AD.

## RESULT

### Identification of differentially expressed gene in AD

**Figure 1:**
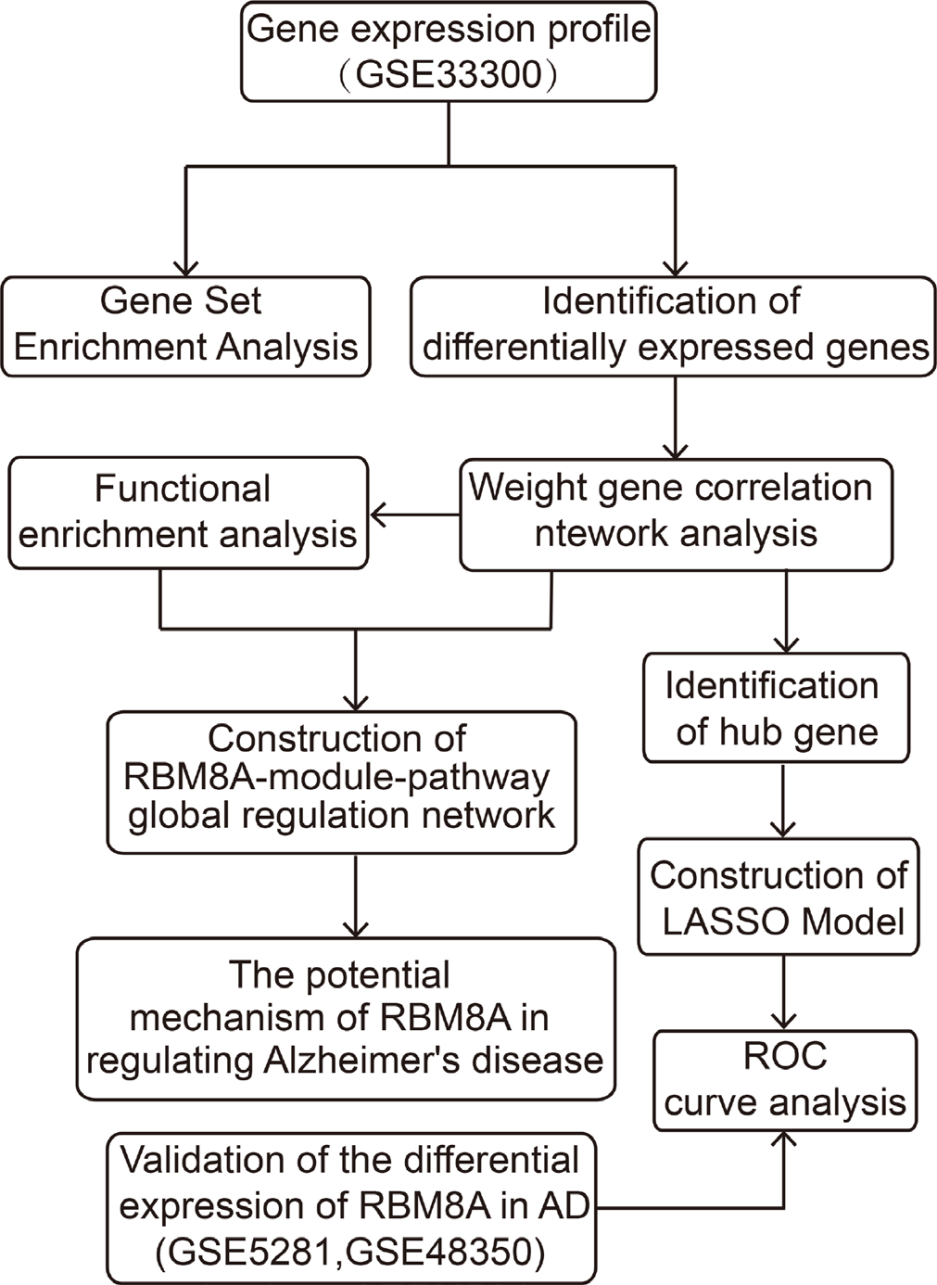
**The workflow of the present study.**

**Figure 2:**
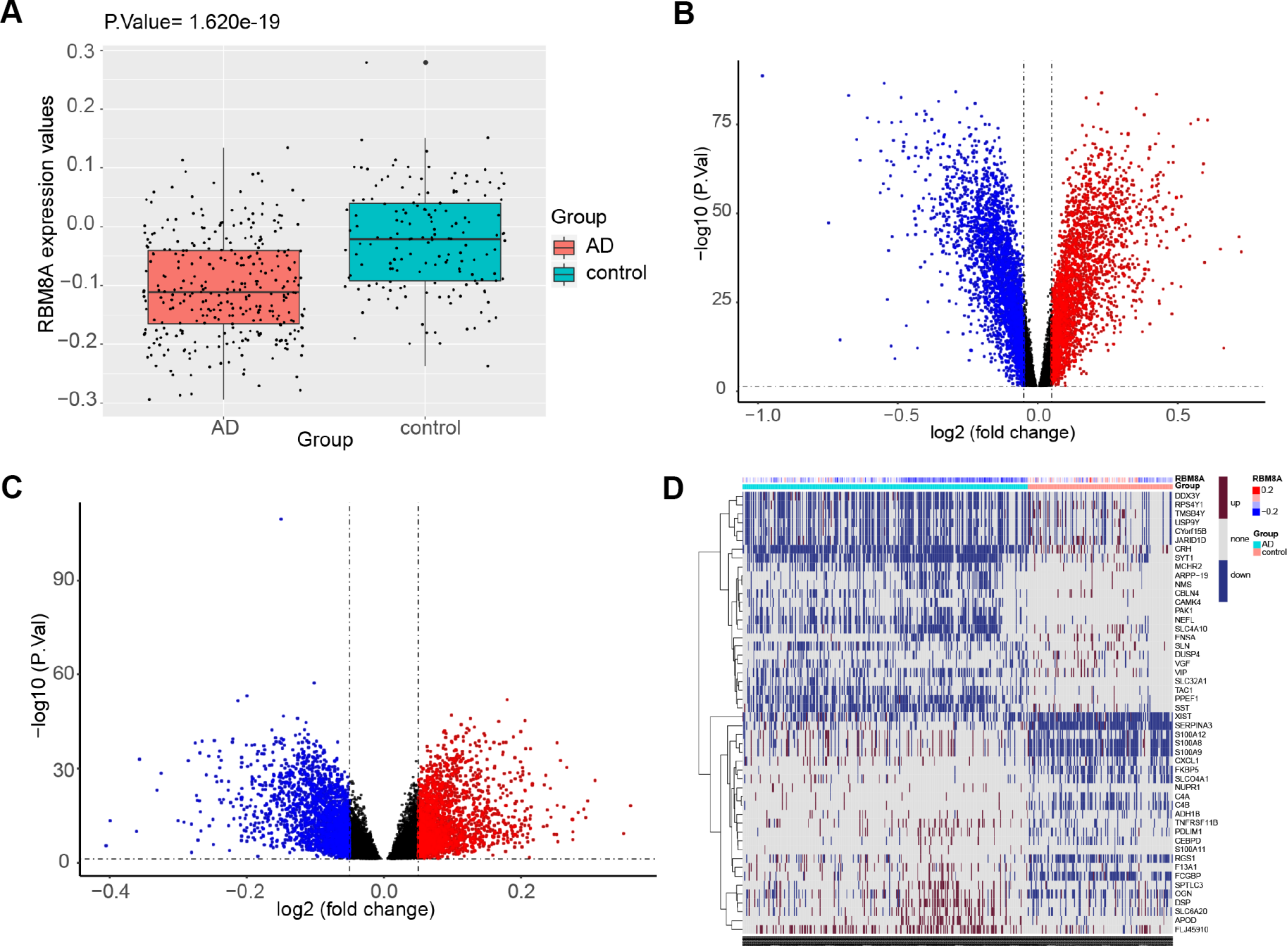
**Differential expression gene analysis.** (**A**) RBM8A is down-regulated in AD (P= 1.620e−19, 310 AD patients and 157 normal people's postmortem prefrontal cortex samples are contained). (**B**) Volcano plot of the AD-control, red represents up-regulated genes, blue represents down-regulated genes, and black represents no significantly differentially expressed genes. (**C**) Volcano plot of the RBM8A-low/high, blue represents down-regulated genes, and black represents no significantly differentially expressed genes. (**D**) A heatmap of 25 most up-regulated and 25 most down-regulated genes.

Using the workflow shown in Figure 1, we found that compared to control samples, RBM8A is significantly down-regulated in AD, P= 1.620e-19 and logFC (fold change) = -0.078 (Figure 2A), suggesting that low expression of RBM8A is associated with AD. There were 15267 DEGs in AD compared to control samples, of which 7346 were up-regulated and 7921 were down-regulated (Figure 2B, Supplementary Table 1). Moreover, compared to RBM8A-high group, there were 13691 DEGs in RBM8A-low samples, of which 7128 were up-regulated and 6563 were down-regulated (Figure 2C, Supplementary Table 2). There were 9186 genes were commonly up-regulated or down-regulated in both AD/control and RBM8A-low/high groups. These genes may be AD-related genes that correlate with RBM8A expression. The 25 most up-regulated and 25 down-regulated genes in AD were visualized in a heatmap (Figure 2D).

### Module associated with AD

**Figure 3:**
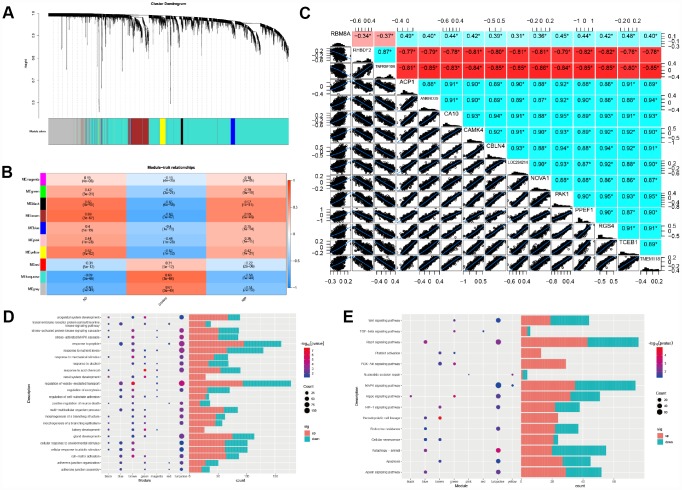
**Weighted correlation network analysis.** (**A**) Recognition module, each module was given an individual color as identifiers, including 10 different modules. (**B**) Correlation heat map of gene modules and phenotypes, the red is positively correlated with the phenotype, blue is negatively correlated with the phenotype. (**C**) The correlation between RBM8A and hub genes, red indicates negative correlation and blue indicates positive correlation (**D**) Biological processes of module genes, the significance of enrichment gradually increases from blue to red, and the size of the dots indicates the number of differential genes contained in the corresponding pathway. (**E**) KEGG pathways analysis of module genes. The significance of enrichment gradually increases from blue to red, and the size of the dots indicates the number of differential genes contained in the corresponding pathway.

To identify the key module most associated with AD, WGCNA was performed using the expression profile of AD-related genes associated with RBM8A level. A total of ten modules were identified (Figure 3A). The brown module is positively correlated with AD (correlation coefficient = 0.69, P = 3E-67; Figure 3B), while the turquoise module is negatively correlated with AD (correlation coefficient = -0.69, P = 3E-66; Figure 3B). According to GS > 0.7 and MM > 0.9, fifteen genes are identified as hub genes (RBM8A, RHBDF2, TNFRSF10B, ACP1, ANKRD39, CA10, CAMK4, CBLN4, LOC284214, NOVA1, PAK1, PPEF1, RGS4, TCEB1 and TMEM118). The correlation analysis shows that RBM8A strongly correlates with hub genes (Figure 3C). Furthermore, module function enrichment analysis shows that brown module genes (Figure 3D) are significantly involved in biological processes related to regulation of vesicle−mediated transport, while turquoise module (Figure 3D) genes are significantly enriched in biological processes of response to peptide, regulation of vesicle−mediated transport, response to nutrient levels, gland development. The brown module (Figure 3E) is significantly involved in Hematopoietic cell lineage and PI3K-AKT signaling pathway, while turquoise module (Figure 3E) is significantly involved in Rap1 signaling pathway, MAPK signaling pathway, Autophagy-animal, Apoptosis and Apelin signaling pathway. In addition, we extract the genes that interact with RBM8A in the modules based on STRING database. According to the pathway which these genes are significantly involved in, we finally constructed an RBM8A-module-pathway global regulation network (Supplementary Figure 1). This network showed that RBM8A indirectly regulates pathways related to AD by regulating interacting module genes.

### Verification of biological processes and key pathways in AD

**Figure 4:**
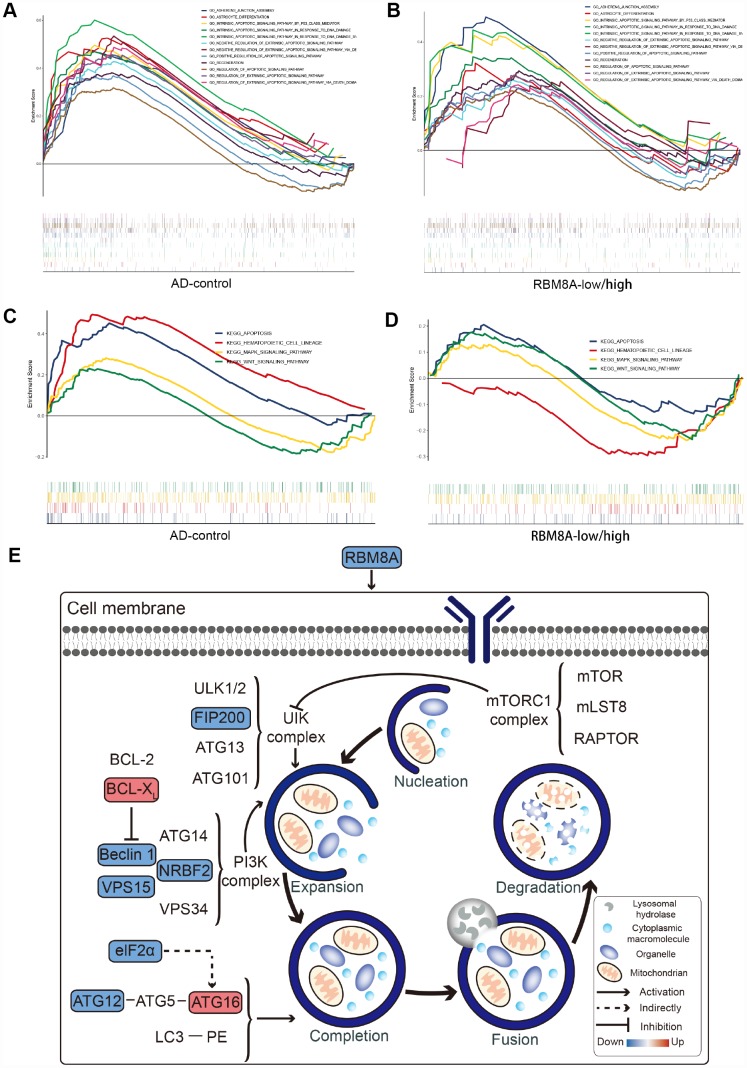
**Result of Gene Set Enrichment Analysis.** (**A**) Biological processes enriched in AD. (**B**) Biological processes enriched in RBM8A-low. (**C**) KEGG pathways enriched in AD. (**D**) KEGG pathways enriched in RBM8A-low. (**E**) Potential mechanism of low expression of RBM8A associated with AD, blue indicates the down-regulated gene and red indicates the up-regulated gene.

Gene Set Enrichment Analysis showed that compared to control samples, biological processes such as adherent junction assembly, astrocyte differentiation, intrinsic apoptotic signaling pathway by p53 class mediator are significantly enriched in AD (Figure 4A). Similarly, compared to RBM8A-high, biological processes, such as Adherents junction assembly, Astrocyte differentiation, Intrinsic apoptotic signaling pathway by p53 class mediator were significantly enriched in RM8A-low (Figure 4B). Moreover, the Apoptosis, Hematopoietic cell lineage, MAPK signaling pathway, and Wnt signaling pathway were significantly involved in AD (Figure 4C). Similarly, apoptosis, hematopoietic cell lineage, MAPK signaling pathway and Wnt signaling pathway are also significantly over-represented in RBM8A-low samples (Figure 4D). Furthermore, we found that low expression of RBM8A affects many modular genes, including FIP200, Beclin 1, NRBF2, VPS15 and ATG12. These genes are involved in key complexes encoding autophagy pathways, which may be a potential mechanism for low expression of RBM8A to promote AD (Figure 4E).

### LASSO model is a potential predictive marker of AD

**Figure 5:**
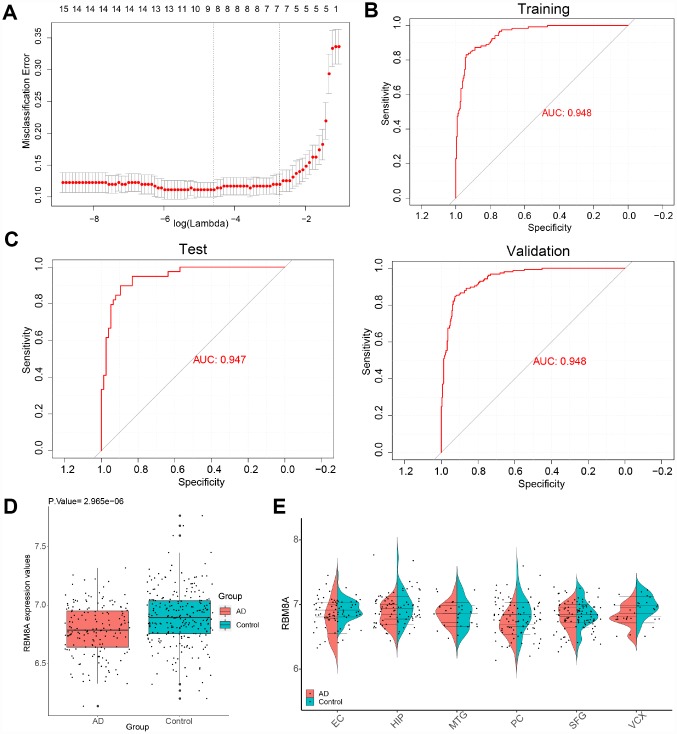
**A model for predicting AD and verification of differential expression of RBM8A.** (**A**) LASSO model. (**B**) ROC curves analysis of train set (GSE33000). (**C**) ROC curves analysis of test set (GSE3300) and validation (GSE5281 and GSE48350) (**D**) RBM8A is down-regulated in AD (P= 2.965e-06, 157 AD patients and 247 normal samples are contained). (**E**) RBM8A is down-regulated in various brain regions. EC: entorhinal cortex, HIP: hippocampus, MTG: medial temporal gyrus, PC: posterior cingulate, SFG: superior frontal gyrus, VCX: primary visual cortex.

We extracted the expression profile of hub genes to construct LASSO model (Figure 5A). Using the LASSO method, 8 genes were identified with non-zero regression coefficients, and the value of lambda.min = 0.04013996. The genes based model index was created as the following formula: index =RBM8A*(-2.38668779488564) + RHBDF2*2.00115481990953 + TNFRSF10B*0.817520478917702 + ACP1*(-3.41028 393841058) + ANKRD39*(-0.279104767589027) + CA10*(-0.988480656766608) + CBLN4*(-0.70290303 609009) + PPEF1*(-1.67911758870231). ROC curve analysis (Figure 5B) indicated that the AUC of the 8-gene-based model was 0.948 in the training set and 0.947 in the test set, which suggesting LASSO model may be used as a biomarker of AD. It was further validated in a test set and a validation set (GSE5281 and GSE48350) with AUC= 0.947 and AUC = 0.948, respectively (Figure 5C). Furthermore, we found that RBM8A is down-regulated in AD (Figure 5D) and in several brain regions (Figure 5E) of patients in GSE5281 and GSE48350. This indicated that RBM8A and its related hub genes were highly associated with AD, and they could serve as biomarkers for further test.

## DISCUSSION

Alzheimer's disease is the most common form of dementia, affecting more than 50 million people worldwide. Despite decades of research, no drugs can effectively treat Alzheimer's disease. In previous studies, we found that RBM8A regulates many risk genes associated with neurodegenerative / neuropsychiatric disorders and many important functional processes that are critical for early neurodevelopment [20]. Therefore, we further explore the regulatory mechanism of RBM8A on AD. In the present study, we first found that RBM8A was generally down-regulated in AD patients, suggesting that low expression of RBM8A may promote AD. According to WGCNA, a total of nine modules associated with AD were identified. Moreover, according to GS > 0.7 and MM > 0.9, fifteen genes were identified as hub genes, including RBM8A, RHBDF2, TNFRSF10B, ACP1, ANKRD39, CA10, CAMK4, CBLN4, LOC284214, NOVA1, PAK1, PPEF1, RGS4, TCEB1 and TMEM118. Among them, DNA methylation changes of RHBDF 2 may play a role in the onset of AD [21], and NOVA1 also plays a significant role in neurological disorders [22], indicating that these genes are strongly correlated with AD. Furthermore, we found RBM8A shows high correlation with hub genes, which suggested that changes in RBM8A expression may lead to changes in these genes.

We further explored the biological processes and pathways related to AD. Functional enrichment analysis showed that modules with a strong correlation with AD significantly involved in biological processes related to response to peptide, response to nutrient levels, regulation of vesicle-mediated transport and gland development, and significantly involved in the Wnt signaling pathway, Rap1 signaling pathway, MAPK signaling pathway, autophagy and apoptosis. GSEA also showed the Wnt signaling pathway, MAPK signaling pathway and apoptosis are enriched in AD and RBM8A-low group. Furthermore, based on the STRING database, we extracted genes that interacted with RBM8A in each module. Because of the interaction, changes in the expression of RBM8A correlates with the expression of these genes. According to the KEGG pathway, using the genes that are enriched in the module with RBM8A, a global regulatory network of RBM8A-module gene-pathway was constructed. Through this network, RBM8A can regulate pathways by regulating module genes directly or indirectly (Supplementary Table 1). Many pathways have been confirmed to relate to AD. For example, down-regulation of canonical Wnt/beta-catenin pathway is associated with AD [23, 24]. The Wnt dysfunction results in Aβ production and aggregation *in vitro*, which promotes AD [25]. Apoptosis was also found to be significantly associated with AD [26]. In our study, we found the Rap1 signaling pathway, MAPK signaling pathway and autophagy were associated with AD. Moreover, mounting evidence has implicated defective autophagy in the pathogenesis of several major neurodegenerative diseases, particularly AD [27–29]. The impairment in the autophagy-lysosome system not only promotes the production of amyloid beta-peptide in Alzheimer's disease (AD), but also interferes with the conversion of other AD-related molecules [30].

Our study identified an interesting finding that the down-regulation of RBM8A expression resulted in the down-regulation of FIP200, Beclin 1, NRBF2, VPS15, eIF2α and ATG12. Among them, FIP200 is a component of the ULK-Atg13-FIP200 complex and is also required for autophagosome formation in mammalian cells [31, 32]. Beclin 1 is a novel Bcl-2-homology (BH)-3 domain only protein [33]. It interacts with several cofactors to regulate the lipid kinase Vps-34 protein and promotes formation of Beclin 1-Vps34-Vps15 core complexes to initiate autophagy. Many studies showed, Beclin 1 dysfunction has been implicated in many disorders, including cancer and neurodegeneration [34]. Inhibition of Beclin 1 function will impair autophagy and promote AD pathology [31]. In addition, studies have shown that NRBF2 (nuclear receptor binding factor 2) is a key component / regulator of the PtdIns3K and is involved in APP-CTFs homeostasis in an AD cell model [35]. ATG12 is a ubiquitin-like molecule that is activated by the E1-like enzyme ATG7, transferred to the E2-like conjugating enzyme ATG10, and ultimately attached to ATG5 [36]. This process is required for the early steps of autophagy [37]. The activity of the conserved ATG12-ATG5-ATG16 complex is essential for autophagosome formation [38]. All these genes are important components of unc-51 like kinase (UIK) complex and phosphatidylinositol 3-kinase (PI3K) complex, and participate in regulating autophagy [39]. Our study reveals the interaction of RBM8A with the autophagy pathway by affecting these six genes, suggesting that low expression of RBM8A may contribute to autophagy disorder and AD by down-regulating these genes.

In addition, the expression profile of hub genes was extracted to construct LASSO model, of which 8 genes were identified with non-zero regression coefficients. Among these 8 genes, some of these genes have been previously reported to be associated with AD. For example, RBM8A is an RNA binding protein that has differential expression in Alzheimer's disease [40], and DNA methylation on RHBDF 2 gene may have a role in the onset of AD [36, 41], the increase of CBLN4 may be a potential therapy for AD [42]. ROC curve analysis showed that in both training set and test set, the LASSO model has a high AUC value and may be served as a biomarker of AD. This is also verified in an independent dataset. Furthermore, we also found that RBM8A is consistently down-regulated in various brain regions of AD patients, which further support that the low expression of RBM8A contributes to the pathogenesis of AD.

Our study proves that bioinformatics analysis can reveal some important insights into molecular pathways underlying AD. However, the potential key pathways and genes are based on bioinformatics tools and molecular experiments should follow to further validate. It remains to be tested to what extent downregulation of RBM8A in AD patients contributes to AD development.

## CONCLUSIONS

Low expression of RBM8A may correlate with the decrease of the components of key complexes (FIP200, Beclin 1, NRBF2, VPS15 and ATG12) in autophagy pathway, which underlie potential novel mechanism contributes to autophagy disorders and AD.

## MATERIALS AND METHODS

### Data processing

In the Gene Expression Omnibus database (GEO, https://www.ncbi.nlm.nih.gov/geo/) [43], we selected three data sets related to AD for analysis. Among them, the GSE33000 based on GPL4372 platform includes 310 AD patients and 157 normal people's postmortem prefrontal cortex samples were used to explore the potential involvement of RBM8A in AD. In addition, the GSE5281 includes 87 AD patients and 74 normal people's brain tissue samples, and the GSE48350 includes 80 AD patients and 173 normal people's brain tissue samples, which both based on GPL570 were used to verify the differential expression of RBM8A in AD patients. The *normalizeBetweenArrays* function in the *limma* package [44] was used to normalize gene expression profiles in GSE33000, while the *justRMA* function in *Affy* package was used to normalize the data in GSE5281 and GSE48350. Moreover, *impute* package (http://bioconductor.org/packages/impute/) was used to supplement missing data, and *ComBat* function in the *sva* package [45] was used to merge GSE5281 and GSE48350 data sets and remove the batch effect. The workflow of the present study was shown in Figure 1.

### Gene set enrichment analysis

Gene Set Enrichment Analysis (GSEA) [46, 47] was used to screen biological process (BP) GO term and KEGG pathways that may be related to AD in GSE33000 datasets. GSEA analysis uses GSEA2-2.2.4 (Java) version for analysis. The c5.bp.v6.2.symbols.gmt and c2.cp.kegg.v6.2.symbols.gmt datasets in MsigDB V6.2 database [48] were used as reference gene sets and GSEA analysis was performed according to default parameters. P < 0.05 was considered significant.

### Identification of differentially expressed genes (DEGs)

Differential expression analysis was performed using the *limma* package [44, 49, 50] in R. We selected the median expression level of RBM8A as the cutoff point to dichotomize patients into RBM8A-high and RBM8A-low expression groups. The DEGs between AD and control, and between RBM8A-high and RBM8A-low were identified using *lmFit* and *eBayes* functions in *limma* package. P < 0.05 adjusted by the false discovery rate (FDR) was considered as significant.

### Weight gene correlation network analysis (WGCNA)

We extracted the expression profile of those genes which are commonly up-regulated or down-regulated in both AD/control and RBM8A-low/high to perform WGCNA [51] in GSE33000. At the first, *hclust* function was used to hierarchical clustering analysis. Then, the soft thresholding power value was screened during module construction by the *pickSoftThreshold* function. Candidate power (1 to 30) was used to test the average connectivity degrees of different modules and their independence. A suitable power value was selected if the degree of independence was > 0.8. The WGCNA R package was used to construct co-expression networks (modules); the minimum module size was set to 30 and each module was assigned a unique color label. In addition, the *clusterProfiler* package [32] in R was used to perform functional enrichment analyses for these functional modules.

### Identification of hub gene and construction of RBM8A-module-pathway network

In WGCNA, gene significance (GS) is defined as the correlation a gene with phenotype. Module membership (MM): MM(i) = cor(x i, ME) is defined to measure the importance of a gene in the module. In this study, a gene with GS > 0.7 and MM > 0.9 was defined as hub gene among the candidate gene modules. In addition, according to the STRING database (https://STRING-db.org/), [53], DEGs that interact with RBM8A were extracted. The correlation of the DEGs interacting with RBM8A and the hub genes was to explore. After that, a RBM8A-module-pathway global regulation network was constructed. Then, *cytoscape* software [54] was used to network visualization.

### Construction of LASSO model and receiver operating characteristic (ROC) curve analysis

Least absolute shrinkage and selection operator (LASSO) has strong predictive value and low correlation and applied to select the best features for high-dimensional data [55, 56]. In order to distinguish AD from control, we extracted the expression profile of hub genes to construct LASSO model by *glmnet* package (https://CRAN.R-project.org/package=glmnet). A model index for each sample was created using the regression coefficients from the LASSO analysis to weight the expression value of the selected genes with the following formula: index = ExpGene1*Coef1 + ExpGene2*Coef2 + ExpGene3*Coef3+. …

The "Coef" is the regression coefficient of gene and is derived from the LASSO Cox regression, and "Exp" indicates the expression values of the gene. Then, GES33000 data set were randomly assigned to the training set (70%) and test set (30%). In order to evaluate the ability of LASSO model to identify AD, *pROC* package [57] was used to conduct ROC curve analysis in the training set and test set.

## Supplementary Material

Supplementary Figure 1

Supplementary Table 1

Supplementary Table 2
