# Supplementary material for: Identification of molecular correlations of RBM8A with autophagy in Alzheimer's disease
Source: Aging (Albany NY). 2019 Dec 9;11(23):11673–85. doi: 10.18632/aging.102571 (PMC6932873; doi:10.18632/aging.102571)
Supplement: Supplementary Figure 1 [file aging-11-102571-s001..pdf]

SUPPLEMENTARY FIGURE

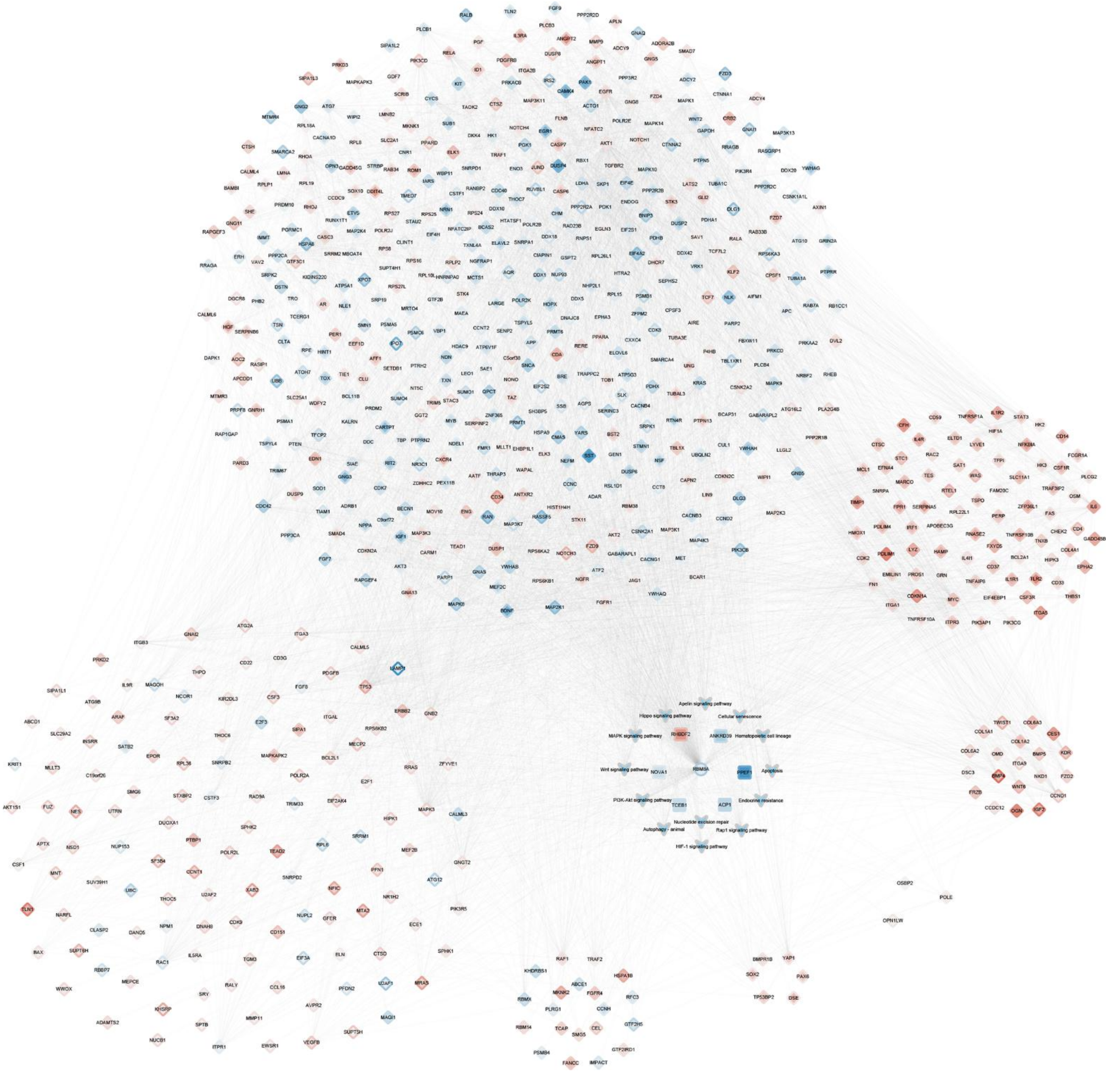

Supplementary Figure 1. RBM8A-module-pathway network. Red represents high expression of genes, blue represents low expression of genes.
